# Remote collection of electrophysiological data with brain wearables: opportunities and challenges

**DOI:** 10.1186/s42234-023-00114-5

**Published:** 2023-06-21

**Authors:** Richard James Sugden, Viet-Linh Luke Pham-Kim-Nghiem-Phu, Ingrid Campbell, Alberto Leon, Phedias Diamandis

**Affiliations:** 1 Department of Medical Biophysics, University of Toronto, Toronto, ON M5S 1A8 Canada; 2 Princess Margaret Cancer Center, University Health Network, 610 University Avenue, Toronto, ON M5G 2C1 Canada; 3 Department of Laboratory Medicine and Pathobiology, University of Toronto, Toronto, ON M5S 1A8 Canada; 4 Laboratory Medicine Program, University Health Network, 200 Elizabeth Street, Toronto, ON M5G 2C4 Canada

**Keywords:** Electroencephalography, Wearable devices, Remote medicine, Diagnostics, Neuropathology

## Abstract

Collection of electroencephalographic (EEG) data provides an opportunity to non-invasively study human brain plasticity, learning and the evolution of various neuropsychiatric disorders. Traditionally, due to sophisticated hardware, EEG studies have been largely limited to research centers which restrict both testing contexts and repeated longitudinal measures. The emergence of low-cost “wearable” EEG devices now provides the prospect of frequent and remote monitoring of the human brain for a variety of physiological and pathological brain states. In this manuscript, we survey evidence that EEG wearables provide high-quality data and review various software used for remote data collection. We then discuss the growing body of evidence supporting the feasibility of remote and longitudinal EEG data collection using wearables including a discussion of potential biomedical applications of these protocols. Lastly, we discuss some additional challenges needed for EEG wearable research to gain further widespread adoption.

## Background

**Fig. 1:**
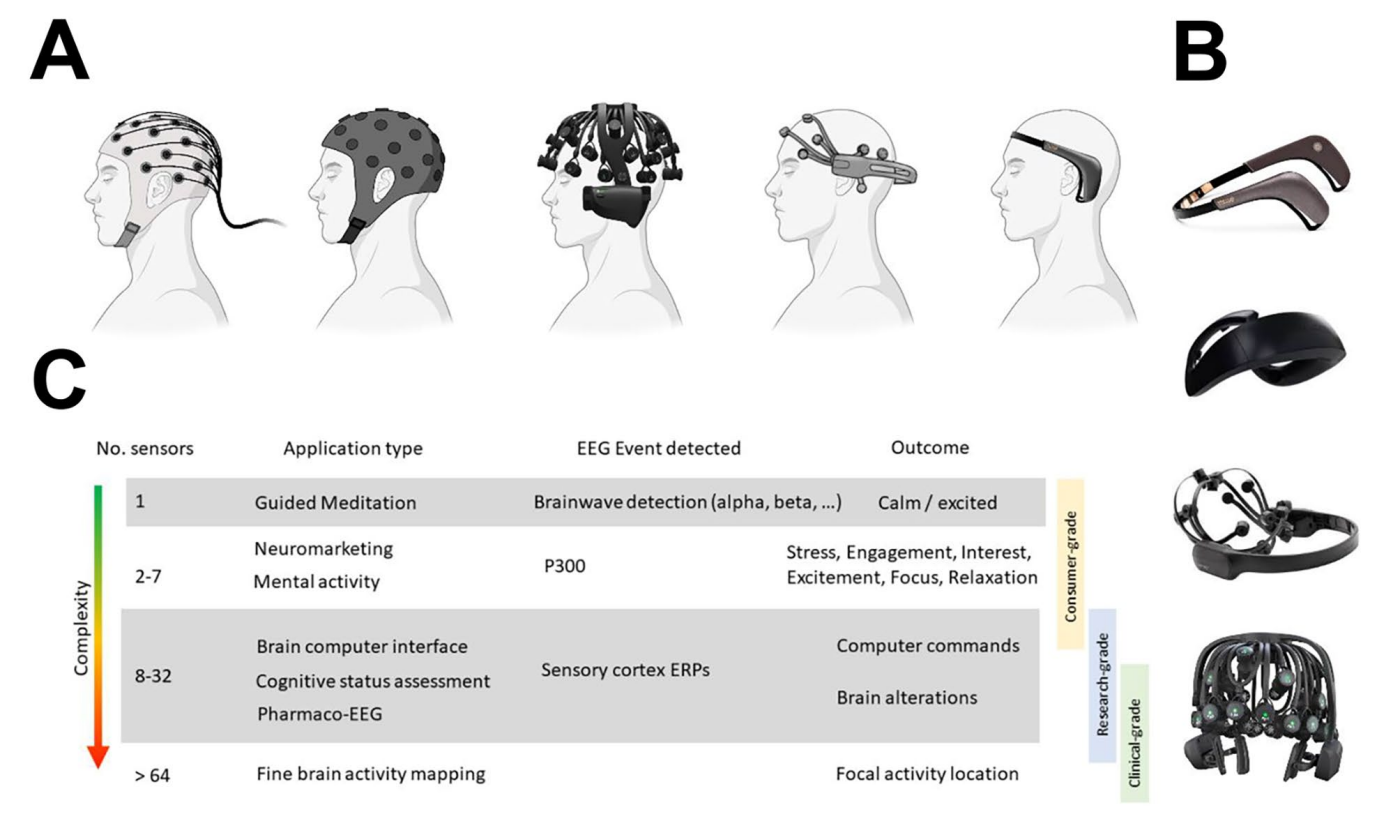
Comparison of EEG data collection hardware. (**A**) shows (left to right): a typical medical EEG setup with a high density of wired electrodes; a research-grade wearable cap with wireless electrodes; a research-grade Quick-32r headset; a research-grade EPOC X wearable with 14 electrodes; and a Muse 2 consumer-grade wearable with 4 recording electrodes. (**B**) shows a column of EEG wearables (top to bottom: Muse 2, Neurosity Crown (Introducing the Crown | Neurosity 2022; This ‘Personal Brain Computer’ Boosts Productivity By Sensing Your Brainwaves And Playing Music From Spotify, 2021), EPOC X, Quick-32r). Examples of additional wearables available on the market (not shown above) include: BrainBit(Wearable EEG headband – BrainBit, 2022) (EEG headband with 4 recording electrodes), Neurosky MindWave (Rieiro et al. 2019) (single electrode EEG wearable), and Neuroon (Liang and Chapa Martell 2018) (EEG wearable sleep mask). (**C**) shows an overview of the: number of sensors, common applications, EEG characteristics, and outcomes that are commonly related (but not limited) to each grade of EEG hardware

Electroencephalography (EEG) is a non-invasive neuroimaging technique whereby a cap containing an array of electrodes is used to measure the electrical activity of the brain. This technique has traditionally been performed in laboratory and clinical settings using high-grade equipment with costs on the order of tens-of-thousands of dollars. This equipment is also complex and time-consuming to set up, requiring a trained technician to install and perform brain imaging studies. Over the past decade, there has been an explosion of interest in consumer-grade EEG “wearables” that are small, lightweight, battery-operated devices with costs typically at least an order of magnitude lower (Fig. 1) (Casson 2019). These wearables come in a variety of forms, such as headsets and headphones, each offering different trade-offs between design (e.g. comfort, obtrusiveness) and capabilities (e.g. spatial resolution, battery life) (Table 1). The cost effectiveness and portability of these wearables has created new opportunities for EEG to be conducted remotely and longitudinally. Current applications of these devices primarily include neuromarketing, brain-computer interfaces (BCIs) (Fouad 2021; Lavermicocca et al. 2018; Peterson et al. 2020), and neurofeedback for focus-related activities (Introducing the Crown | Neurosity 2022; This ‘Personal Brain Computer’ Boosts Productivity By Sensing Your Brainwaves And Playing Music From Spotify, 2021) such as meditation (Hunkin et al. 2021; Millstine et al. 2019), and cognitive load (Huang et al. 2020; Medeiros et al. 2021). While the literature on these applications is still in its infancy, even less is known about the potential for EEG wearables to be used in biomedical applications such as the remote monitoring and detection of neurological disease. Here, we discuss significant milestones that have been made toward this aim, key pre-requisites that still need to be addressed, and propose some long-term milestones for the field at large.

**Table 1:** Forms of EEG wearables. This table summarizes some of the key trade-offs facing the most common forms of EEG wearables.

Wearable Category	Key Advantages	Notable Compromises	
Headset	Better scalp coverage, more emphasis on data quality	Challenging for participants to setup independently	
Headband	Comfortable, design suitable for use at-home	Moderate scalp coverage (sagittal plane) and less emphasis on data quality	
Tattoo (adhesive sensor)/Behind the ear sensor	Very discrete (< 1inch), battery life good for passive monitoring	Most restricted scalp coverage	
Headphones/Earbuds	Minimally obtrusive, design suitable for use anywhere	Headphones have moderate scalp coverage (coronal plane), earbuds have highly limited coverage (around ear)	

## Requirements

### Data quality

**Fig. 2:**
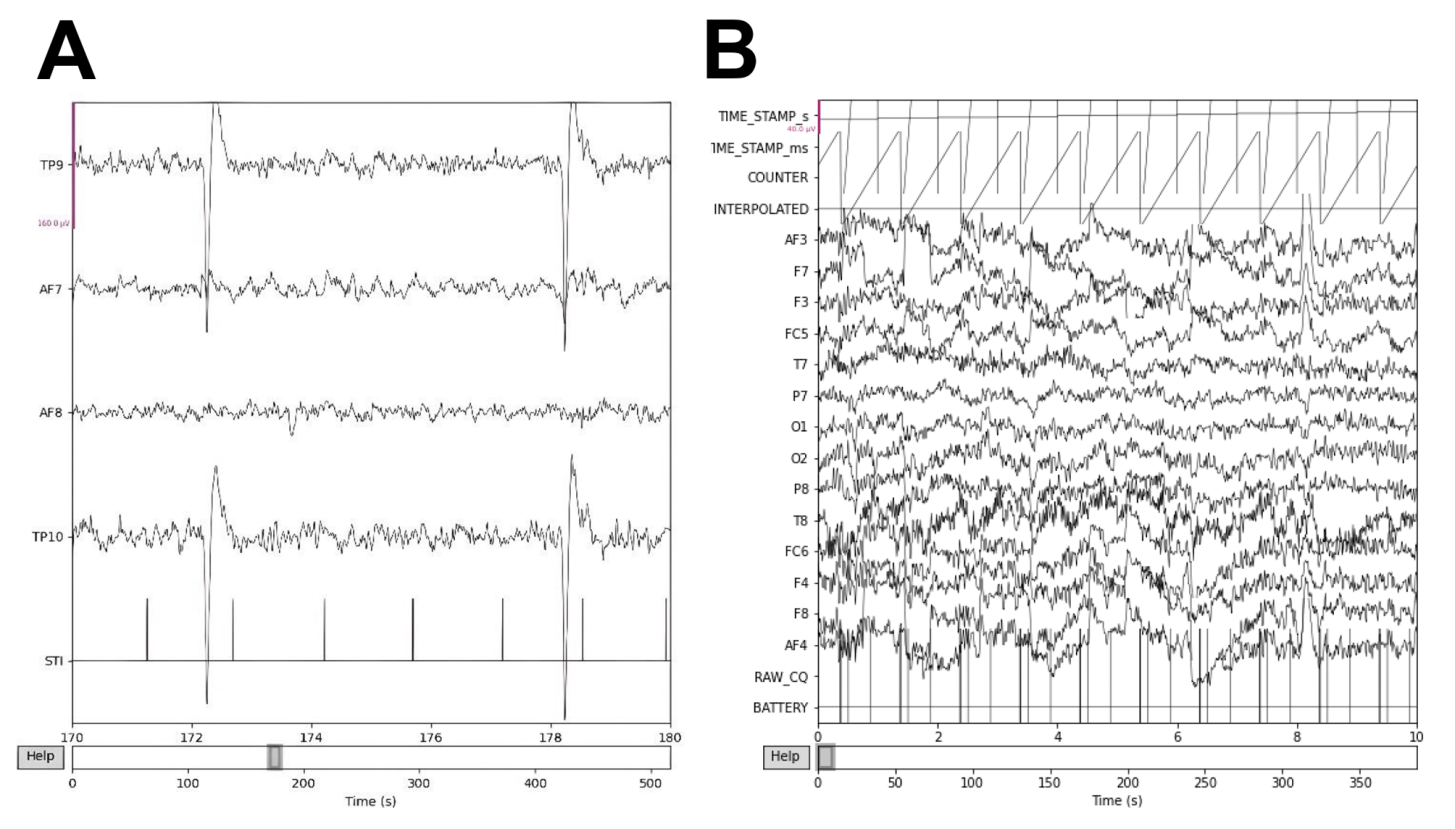
Examples of raw EEG data collected from consumer-grade wearables. (**A**) Raw EEG data collected from a consumer-grade 4-electrode Muse 2 wearable. X axis represents time and Y axis shows electrode labels. AF and TP are labels for the data collected by anterior-frontal and temporoparietal electrodes, respectively. The scale is shown by the red line representing 160 microvolts of amplitude. Large spikes are likely to be a result of ocular and muscular artifacts. (**B**) Raw EEG data collected from a research-grade 14-electrode EPOC X wearable. Time is shown on the X axis and electrode labels on the Y axis (international 10–20 system)

**Fig. 3:**
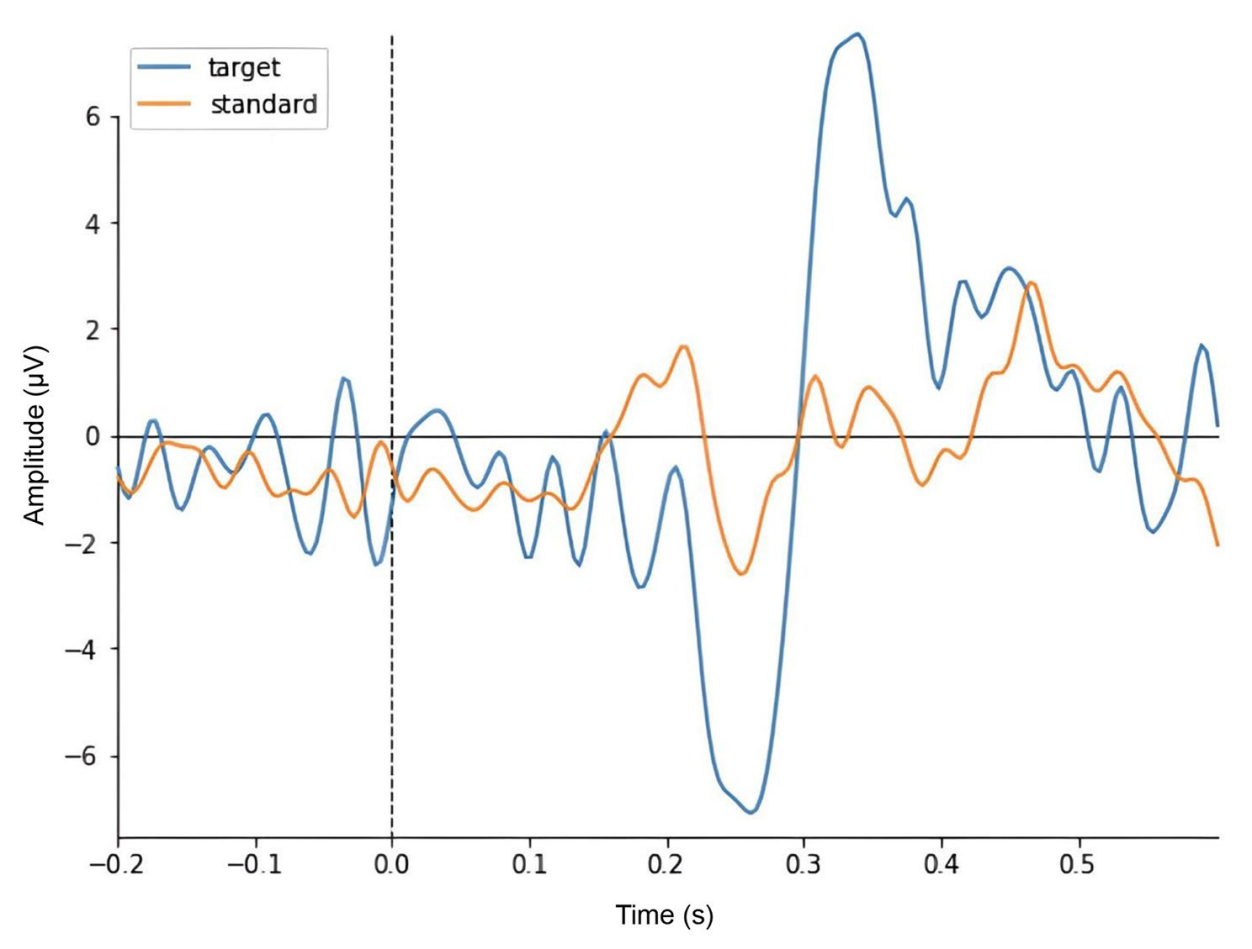
Example of a P300 event-related potential collected from an oddball task. The X axis shows the time in seconds with 0 s coinciding with stimuli presentation (dotted line). Y axis shows the EEG signal amplitude in microvolts. Blue and orange lines represent an average of EEG waves evoked by rarely occurring target stimuli (e.g. visual or auditory cue) and an average of EEG waves evoked by commonly occurring standard stimuli, respectively. Observing differences between the target and standard data reveals two characteristic waveforms, a positive spike at 300 ms (P300) preceded by a negative spike at approximately 200 ms (N200)

To incorporate consumer-grade EEG wearables into remote and “operator-free” research protocols, a key milestone would be the demonstration of the ability to collect neurocognitively informative data from raw EEG recordings in traditional research settings (Fig. 2). Generally, free-running EEG measurements that do not involve external stimuli can be utilized to analyze basal cognitive networks by observing the brain’s resting-state activity. However, these measurements are typically combined with stimuli-paired recordings to facilitate the analysis of time-locked data known as event-related potentials (ERPs). A commonly used example of ERP analysis is the oddball test, which can be administered visually or auditorily. During this test, subjects are presented a random series of common (frequency: 80–90%) and less common (frequency: 10–20%) visual or auditory events. The less common events often elicit characteristic ERP waveform reflexes from the subject’s unconscious novelty seeking system. Averaging these events over multiple presentations can reveal several characteristic ERPs, including the P300 and N200 waveforms, which are positive and negative spikes in activity occurring approximately 300 and 200 milliseconds after stimulus presentation, respectively (Fig. 3) (Krigolson et al. 2017; Squires et al. 1975). These EEG waveforms are dependent on intact levels of cognition, and alterations in their timing and amplitude are often associated with various intra-cranial pathologies, including depression, neurodegeneration, and addiction (Boutros et al. 1995; D’Arcy et al. 2003; Duncan et al. 2003; Zhou et al. 2019).

These waveforms were successfully imaged by Krigolson et al. in a seminal 2017 paper that demonstrated that Muse 2 wearables (~$250 device available on Amazon) and a clinical Brain Vision system produce nearly identical P300 and N200 waveforms in 60 healthy participants (Krigolson et al. 2017). These findings were further supported by the same group in 2021 where significant correlations were found between perceived cognitive fatigue and the combination of EEG and ERP-derived features in 1000 participants (Krigolson et al. 2021). While these papers represent a cornerstone achievement for EEG wearables, both studies still involved the presence of trained researchers during data collection. In contrast to the serial data collection protocol of the 2017 Krigolson paper in which the simple Muse devices were benchmarked to subsequent higher density EEG headset recordings, Kutafina et al. performed simultaneous collection. In this study, participant resting state activity was measured with both the Emotiv EPOC X wearable and a clinical system known as the Brain Quick Plus Evolution by Micromed, to assess the simultaneous correspondence between their neighboring electrodes (Kutafina et al. 2020). This study found a promising time-domain correlation between the two systems, suggesting that wearables could have the potential to replicate clinical findings. However, no significance values were reported on these correlation metrics and there are several known limitations of simultaneous recordings such as the assumption that neighboring electrodes should produce highly correlated waveforms despite their slightly different locations on the scalp (Casson 2019). Conversely, Badcock et al. were able to validate the Emotiv EPOC by performing simultaneous recordings with a research-grade Neuroscan system where they observed no significant differences in their auditory ERP amplitudes and latencies in adults (Badcock et al. 2013), and very few differences when repeated in children (Badcock et al. 2015). By performing simultaneous recordings with the EPOC flex saline and the same Neuroscan system, the same group also validated its ability to collect reliable auditory and visual ERPs, and to detect changes in alpha oscillations (Williams et al. 2020). In contrast, Duvinage et al. observed significantly worse performance in a P300 BCI task when using the Emotiv EPOC in comparison to a medical device (Duvinage et al. 2013).

However, it is crucial to highlight that these studies rely on EPOC systems which are considerably more complex for untrained users and require expensive software subscriptions compared to other brands of wearables — both of which present challenges for remote, consumer applications. A much simpler (single-electrode) and less-expensive system known as the Neurosky MindWave was similarly assessed by Rieiro et al. using simultaneous recordings to compare it with a medical-grade EEG device during resting states and virtual-driving tasks. Their analysis revealed significant correlations in signal quality between the wearable and medical-grade systems, such as with blink detection rate and substantial signal stability despite having increased noise. Although, the use of a single electrode in the Neurosky MindWave limits its spatial resolution and ability to address more heterogeneous and asymmetric brain states and lesions (Rieiro et al. 2019). Comparative studies between EEG wearables, whether conducted sequentially or simultaneously, are limited because of the challenges in two devices collecting data concurrently from the same location. In the Muse 2 study (Krigolson et al. 2017), attempts to detect canonical ERP patterns meant that a ground truth measurement was not strictly required. However, with simultaneous recordings such as in the later EPOC X study by Kutafina et al. (2020), there was an *a priori* assumption that neighboring electrodes should have highly similar data (Kutafina et al. 2020). Despite this limitation, performing simultaneous recordings can still provide the ability to discern whether observed differences are attributable to variations between the systems or disparities in the brain’s state at different time points. More specifically, it eliminates confounding factors such as varying levels of fatigue or differences in habituation from exposure to task-related stimuli (Badcock et al. 2013).

Although many of these studies have focused on reproducing electrophysiological signals from well-characterized tasks, such as validating ERPs or resting state data, it is also important to further assess EEG wearable data quality for biomedical applications. For instance, validating wearable EEG data quality for sleep can help augment the accuracy of detecting sleep disorders and evaluating the effectiveness of treatments remotely. In pursuing this, one study by Nakamura et al. performed in-lab simultaneous sleep recordings with a custom single-ear EEG and a standard clinical scalp EEG and found substantial agreements when manually scoring and comparing their hypnograms (Nakamura et al. 2017). Although this supports that accurate and remote EEG recordings with a minimal number of electrodes can be used to feasibly track sleep parameters, further studies on the data quality of commercially available wearables with a higher number of electrodes are required which would enhance the capability for more comprehensive monitoring of brain activity. In 2020, the Dreem EEG consumer headband with 5 electrodes was tested overnight at a sleep laboratory against simultaneously collected standard clinical polysomnography which revealed low mean percent errors between their measurements of relative spectral power. Moreover, they found strong agreements in their ability to detect heart rate, breathing frequency, and respiratory rate variability, as well as in comparisons between the headband’s automatic deep-learning sleep staging classifications to manual classifications conducted by sleep experts on the polysomnograms (Arnal et al. 2020). Overall, the validation of wearables for collecting general EEG data in sleep is promising although further studies with a greater focus on monitoring sleep disorders are required.

Currently, the pathology most widely studied using EEG wearables has been epilepsy due to the fact that traditional EEG studies are well-established for this disease. It has been found that EEG wearables have sufficient data quality to resolve the features of epilepsy for seizure detection (Baum et al. 2022; Glaba et al. 2021; Mckenzie et al. 2017; Neumann et al. 2019). For example, a 2020 in-lab study compared a 2-channel wearable known as Neury with simultaneously collected medical-grade EEG in patients with the epileptic symptom of continuous spike-waves during sleep. Their analysis revealed robust associations between the two systems’ quantitative spike measurements, and side-by-side comparisons between device EEG background rhythms, spike activities, and active ictal recordings also demonstrated strong qualitative concordances (Carvalho et al. 2020). Although non-EEG wearables have displayed considerable potential for seizure prediction (Karoly et al. 2020; Stirling et al. 2021a, b), there is only one pilot study using wearable EEG information where various wearables were combined into a multi-modal analysis (Zambrana-Vinaroz et al. 2022). Additional research is needed to determine if wearable EEG data contains more nuanced signals like subtle epileptic activity and signatures of progression, which could potentially extend wearables’ capabilities to other neurological diseases. For example, most patients with brain tumors exhibit complex but informative EEG signatures at the time of diagnosis (Small et al. 1961). Therefore, it would be valuable to investigate if wearable devices can detect these subtle variations as well as monitoring for any changes in these patterns over time, particularly in the early stages of disease before symptoms become apparent (Samuel et al. 2021).

Besides comparing data similarity to higher-grade devices, it is essential to consider the practical design elements of EEG wearables as they bear important implications for at-home usability for patients. To elucidate these differences in design between devices, Radüntz et al. compared seven different mobile EEG systems with respect to ease-of-use, wearing comfort, visual appearance, setup time, and maximum possible wearing duration. They found large differences in usability between wet and dry electrodes, which can substantially impact adherence to daily use primarily due to the inconvenience of setup (Radüntz and Meffert 2019). Previous studies have also shown that dry electrodes are more likely to provide weaker signals in the short-term but are more stable over longer durations compared to wet electrodes which rely on gradually dissipating gels and saline solutions (Hinrichs et al. 2020). This suggests that for remote studies, researchers need to carefully consider the trade-offs between device usability, comfort, and the expected signal quality.

Ultimately, the culmination of these studies has yielded promising protocols for gathering neurocognitively informative EEG data using consumer-grade wearables. However, it is crucial to recognize that while this milestone is significant, it differs from the objective of remotely acquiring data from patients’ homes, which necessitates further formal testing to ensure reliability and validity.

### Appropriate software

One of the most significant challenges in remote data collection, even with the plethora of suitable EEG devices, is the need for specialized software to synchronize the wearable to a computer collecting data while guiding subjects through neuro-cognitive tasks. For some study designs where macroscopic frequency-domain features are used, such as in many meditation studies, only free-running data collection is required. However, for study designs requiring time-domain responses to stimuli, such as ERPs, collecting data remotely is a considerable challenge. To address this challenge, software that can accurately and precisely timestamp EEG data in real time in relation to stimuli is required (Williams et al. 2021), while also maintaining an easy-to-use interface to facilitate independent data collection from patients at home.

Several investigators have developed software for remote data collection. For example, Li et al. created an EEG acquisition system that allows a Raspberry Pi chip to connect EEG electrodes to a computer using Python (Li et al. 2018). While they managed to produce an effective low-cost system that achieved high signal quality, their hardware configuration included wires and silicon chips that are likely too complex for the primary subjects of clinical studies who may have low technological fluency (i.e. older subjects suffering from neurodegeneration). To alleviate the dependency on wires, Memon et al. proposed an acquisition system for a 4-channel configuration of the OpenBCI wireless EEG system for brain-computer interface applications (Memon et al. 2018). While succeeding with a wireless system is a considerable milestone, the numerous components and attachments required to setup this device could still be too complex for many patient populations and may only be practical and appropriate for lab environments. Ideal software systems should be compatible with commercially available EEG wearables since those devices have been created with careful consideration for design and usability. Prospectively, these systems should be sufficiently modular to integrate new hardware as they are released, and publicly available for external validation and use.

**Fig. 4:**
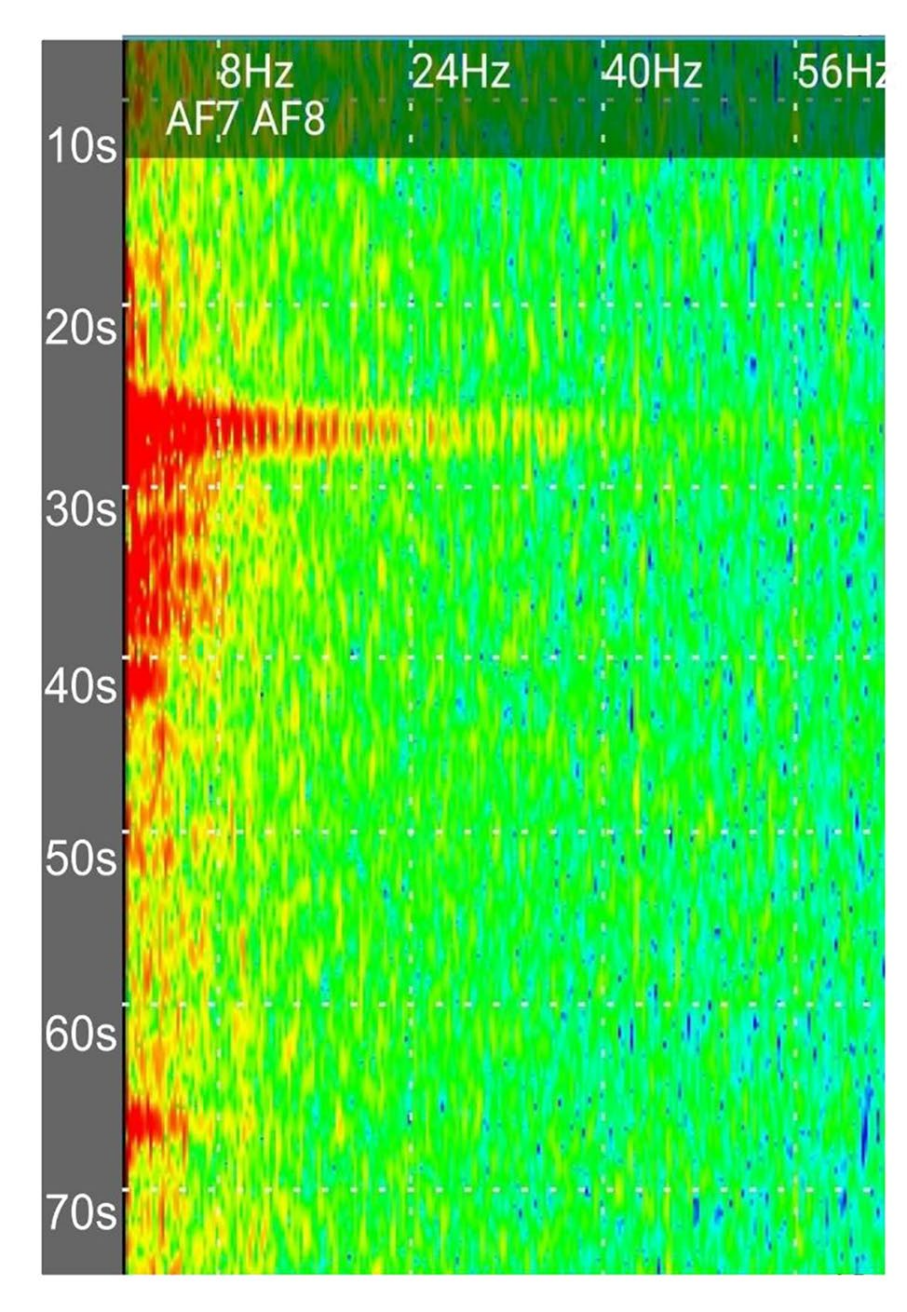
Spectrogram data collected from an EEG consumer wearable. This shows spectrogram data from 2 electrodes of a Muse 2 wearable connected to the Mind Monitor smartphone app (Mind Monitor 2022). Frequencies of EEG waves are shown on the horizontal axis. Time is on the vertical axis starting after zero seconds from the top. Power of the EEG signal for each frequency is encoded by color. Note that color bars are not available in Mind Monitor software so the spectrogram should be interpreted qualitatively. Colors are from high to low power in the following order: red, orange, yellow, green, cyan, blue. A spike in power can be observed earlier in the recording from approximately 0–24 Hz, likely due to an ocular or muscular artifact

Lastly, the restricted access of proprietary programming languages such as MATLAB used by previous groups presents a barrier to public use and researchers who are looking to replicate data (Krigolson et al. 2017, 2021; Kutafina et al. 2020). For example, the collection of proprietary metrics rather than raw EEG data can reduce transparency, reproducibility across devices and limits opportunities for analyses of other widely studied phenomena such as ERPs, or changes in spectrographic activity (Fig. 4). However, this can be resolved with open-source programming languages such as Python, or EEGLAB (Cao et al. 2019) which are likely to grow in popularity as a result. For instance, Cherep et al. (2019) created custom Python software to collect data using a nine-electrode commercial EEG wearable (Cherep et al. 2022). Likewise, our lab is developing a Python-based software prototype to address many of the aforementioned limitations regarding device modularity, public-availability, and ease-of-use. Another issue we have encountered is the dropping of connections, where the device stochastically disconnects from the system, thus, terminating data collection. Similarly, while our efforts centered on ensuring that our software is compatible with various devices, such as the Muse 2 and Muse S, certain systems like the EPOC X require a third-party subscription software to facilitate integration, resulting in increased complexity and reduced shareability of the workflow. This implies that the level of accessibility for users may vary depending on the specific hardware, as it will depend on the availability of open-source compatibility layers between Python and the hardware. Careful selection and promotion of open-source software may therefore be critical to allow studies to be easily reproduced and extended.

## Promises

### Remote acquisition

EEG wearables offer a significant advantage in that they have the potential to collect data remotely. This feature enables the collection of large datasets in parallel, outside of highly controlled environments that may also introduce confounding variables. Many examples of progress towards at-home data collection have been championed by sleep studies. One of the first examples was Liang et al. which used commercial wearables at home to compare EEG to the wristwatch wearable “Fitbit” for tracking sleep onset and duration (Liang and Chapa Martell 2018). They found that the Neuroon (an EEG wearable sleep mask) was able to measure more sleep parameters than the Fitbit as confirmed by a 2-channel clinical sleep measurement system known as the Sleep Scope, which served as ground truth. An example of a more technically focused study was Debellemaniere et al. which successfully collected ERPs at home as part of an interventional sleep study (Debellemaniere et al. 2018). This group was able to modulate ERPs in response to an auditory stimulus and achieved statistically more slow-wave sleep in comparison to a sham treatment. Other successful remote sleep studies have been conducted by several groups: Lunsford-Avery et al. validated the ability to remotely record sleep activity using a wearable EEG (Zmachine Insight + device - General Sleep Corporation) and used correlation to wrist-worn actigraphy as a metric for success (Lunsford-Avery et al. 2020). Rocknathan et al. performed a similar protocol by comparing an exposed-wire EEG system with wristband actigraphy devices and found that EEG parameters better correlated with subjective reports of sleep quality but failed to find any reliable effect of a white noise intervention due to issues with data collection (Rocknathan et al. 2018).

Overall, sleep studies would highly benefit from collecting data at home since sleeping in an unfamiliar environment (i.e. the sleep lab) is a known source of confounding to many features of sleep (Byun et al. 2019). Other researchers have also deployed EEG wearables remotely to collect awake-state neurocognitive information. For example, Barbey et al. (2022) used a research-grade device to demonstrate that EEG P300s can be collected at home over a span of several weeks (Barbey et al. 2022). They achieved a canonical P300 in younger subjects but only an ambiguous waveform for elderly subjects which suggests that technological literacy may be a confounding variable for these remote studies. While this result is a promising milestone for remote data collection, the authors did not include objective analyses of these waveforms to demonstrate significance. Using the consumer-grade Muse 2, Hunkin et al. was able to successfully collect remote EEG data from over 20 participants to study psychological trait-mindfulness. They found significant correlations between proprietary Muse software metrics, and both subjective and objective measures of focus such as psychometric questionnaires and the breath-counting task, respectively (Hunkin et al. 2021).

**Fig. 5:**
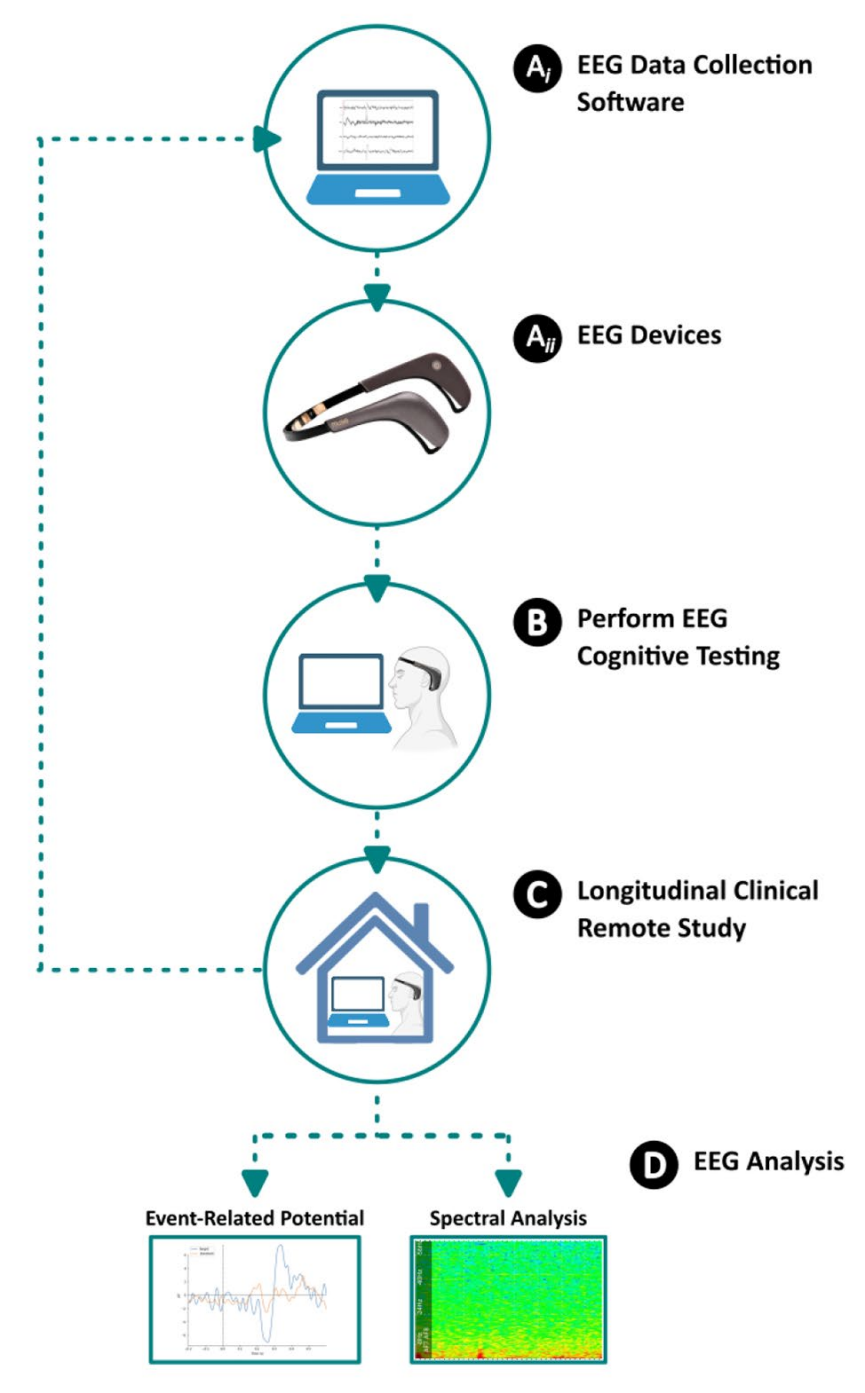
Methodology for remote data collection with consumer EEG wearables. A potential workflow for a remote data collection protocol. First, a laptop or other smart device with software (**A**_**i**_) that can connect to and receive data from an EEG wearable (**A**_**ii**_) is required. A participant would then attend an initial in-lab demonstration on how to navigate the software to simultaneously perform EEG recordings with cognitive tests (**B**) of interest. The devices are then taken home for remote longitudinal data collection (**C**) with sessions as frequent as for example, weekly, bidaily, or even daily. Once collection is completed, devices are returned to the lab for analyses of EEG features, such as event-related potentials or changes in power bands and spectrographic data (**D**). Remote transfer of data is also a potential additional feature with increased data encryption and security measures

To date, our lab’s experiences with remote data collection have revealed additional challenges that may hinder the participants’ abilities to reliably collect data. Successful collection requires an understanding of how to use the data acquisition software installed on a laptop in tandem with the EEG wearable (Fig. 5). Although participants receive an initial demonstration in the laboratory and an instruction document to take home, those who suffer from diseases affecting mobility and/or cognition or who are less technologically literate are more likely to encounter difficulties in completing the remote protocol independently. In these situations, an assistant such as a caregiver or family member would be required to help guide the participant. Thus, an overall priority should be for simple and intuitive software interfaces and supplementary instructions to augment its accessibility.

### Longitudinal acquisition

EEG wearables offer the advantage of expediting the collection of EEG data repeatedly and over a longer duration with fewer practical limitations. Traditional EEG methods necessitate a skilled technician to operate the imaging system, multiple lab visits which can be inconvenient, and high hardware and operation costs that are often too prohibitive to conduct multiple data collection sessions. Due to the ease-of-use, portability, and affordability of consumer-grade wearables, subjects could theoretically collect daily data to explore how EEG signatures evolve across different routine activities, occupational settings and time. A prototypical example of how longitudinal data is traditionally accomplished is provided by Saggar et al. In this study, EEG data was collected from three timepoints (pre, during, and post) across a three-month meditation retreat. This allowed them to demonstrate that an intensive meditation regimen had reproducible effects to individuals’ alpha frequency (Saggar et al. 2012). Another similar example is Lanzone J. et al., where a test-retest study design was performed to show that EEG could be used to track longitudinal changes in stroke recovery, with two time points, two months apart (Lanzone et al. 2022). While studies like these can provide valuable information about changes in brain states, the temporal resolution is limited, which does not allow for making robust claims about the dynamic evolution of EEG signatures. With more frequent EEG collection (weekly, bidaily, or daily) researchers could gain deeper insights into various conditions, including disease pathogenesis, or how meditation and other neurocognitive exercises affect the brain over time. As such, this is likely going to be one of the major differentiating features of wearable EEG devices.

## Biomedical Application

### Epilepsy

From a health science perspective, the cardinal goal of wearable EEG research is to prove the biomedical utility of its collected data. Currently, many wearable EEG studies have focused on epilepsy as a model disease due to its well-defined EEG characterization and high signal-to-noise ratio (Brinkmann et al. 2021; Rosenow et al. 2015). For example, McKenzie et al. performed one of the first studies on biomedical applications using EEG wearables and observed some potential for a smartphone-linked EEG headset to monitor epilepsy compared to standard EEG. However, this study was simply a proof-of-concept and thus did not assess the remote or longitudinal objectives of wearable EEG research (Mckenzie et al. 2017). Neumann et al. performed a comparison and determined that although their portable Fourier One EEG system generates lower quality signals than clinical data, diagnostic ability in patients with epileptic symptoms was not significantly reduced (Neumann et al. 2019). The same group further confirmed the feasibility of collecting data with this portable EEG system at home (Baum et al. 2022). The potential for biomedical applications with EEG wearables was further supported by Glaba et al. which demonstrated that these portable devices can effectively correct for motion artifacts (Glaba et al. 2021). This work resulted in improved real-time seizure detection algorithms, thereby bringing epilepsy detection by EEG wearables one step closer to real-world implementation.

The motivation for the remote monitoring of epilepsy is strong since it can be difficult to detect if a subject does not express any symptoms during their relatively short clinical assessments. Furthermore, having a device that can detect seizures and relay this information to a smartphone that could then alert consented family members of distress has significant implications. However, despite the promising potential for remote epilepsy monitoring, this condition involves a macroscopic electrical discharge that should be easily detectable by any electrode system such as EEG. On the other hand, the application of wearable EEG devices to other diseases may require the detection of more subtle electrophysiological patterns and may require more sophisticated hardware, which may never achieve the same level of spatial and temporal precision as research-grade devices.

In support of broader applications, there are many other neurological diseases that have been recently studied using EEG wearables. For example, Mercado-Aguirre et al.’s study with the consumer-grade Emotiv EPOC demonstrated its ability to detect literature-supported differences in ERP profiles between healthy children and those with ADHD (Mercado-Aguirre et al. 2019). Furthermore, Cao et al. used wearables to successfully identify changes in EEG signatures in response to ketamine treatment for patients with depression, although in a non-remote and non-longitudinal setting (Cao et al. 2019). Additionally, Lin et al. developed a custom-made 8-channel EEG headset that found in-lab differences in ERP characteristics between Parkinson’s disease patients with and without impulse control disorders (Lin et al. 2021). Lastly, a study by Ferster et al. developed an algorithm that can more accurately track phase changes in real time to maximize clinically beneficial in-phase stimulation of slow waves during deep sleep in older adults or patients with neurodegenerative diseases (Ferster et al. 2022).

The successful application of EEG wearables in various neurological diseases is a promising sign that they could offer biomedical insights. So far, these results have been context-specific and were designed to perform an analogous function to replace more expensive EEG systems. The next milestone will be to determine whether EEG wearables can offer something completely novel to the field of EEG. For example, it will be of interest to see if generating large longitudinal EEG datasets can identify longitudinal biomarkers of either disease progression or treatment response, which would be logistically challenging with more traditional approaches. To investigate such a research question, the unique advantages of EEG wearables for performing large-scale data collection over extended periods and for large sample sizes will be required. If wearables prove to be biomedically informative, they could eventually be utilized in a similar manner as Holter monitors for heart monitoring. A patient could then be given an EEG wearable to perform monitoring at home and their physicians could analyze the collected data to make informed clinical decisions. If accurate biomarkers are identified, this could potentially be valuable for screening for neurological diseases at an early stage or tracking disease responses to treatments or interventions.

## Remaining Challenges

### Multimodal wearable data

While EEG wearables are unlikely to ever provide the same data quality as higher-grade systems, there are opportunities to augment their data with other forms of wearables such as wrist-worn, finger ring, or clothing-based devices. These other wearables can provide complimentary information such as movement, heart rate, and respiration which could improve our ability to understand subjects’ physiological states (Zambrana-Vinaroz et al. 2022). Before this concept can be used for any practical applications, several requirements need to be met. For example, the appropriate data collection software(s) would have to be able to precisely integrate incoming data from different sources (which potentially have different sampling rates or transmission delays).

### Optimization

While there is a growing body of evidence that EEG wearables can provide insightful data, we note the large disparities in usability between wearable systems. Each wearable system faces a trade-off between capability, ease-of-use, and comfort. For example, our lab has found that while the Neurosity Crown covers all lobes of the brain, it suffers from a short battery life. In contrast, our participants report that Muse systems are lightweight and easy-to-use, but they only capture data from a two-dimensional sagittal plane. One exciting avenue for future work will therefore be to integrate reports of user feedback into the development of new EEG wearables that optimize design elements (usability, comfort, and style) against technological considerations like electrode count, battery life, etc. We anticipate that, as more research becomes available, future headsets will be increasingly specialized to perform specific tasks by placing electrodes optimally to detect a brain state such as pathology. By determining the optimal locations, developers can use the minimum required electrodes for successful detection to preserve the streamlined design. This would also allow them to include tailored design considerations for clinical populations that may face challenges such as inflammation from surgery.

## Conclusion

Wearable EEG devices provide a unique opportunity to scale and transition non-invasive brain monitoring beyond the limited number of available medical centers. This is analogous to the recent breakthrough devices such as the Apple Watch and Fitbit that have enabled heart rate monitoring and arrhythmia detection for the general public (Perez et al. 2019). The ability to carry out EEG data collection remotely offers many advantages including cost-effectiveness, increased accessibility for population and at-risk screening, and the novel ability to study longitudinal changes across various physiological and pathological states. Despite this promise, there are still many uncertainties that need to be resolved including potential improvements in signal acquisition while balancing independent operation and costs. The actionability of these changes once identified would also need to show merit.

## Data Availability

Not applicable.

## List of Abbreviations

EEG: Electroencephalography
ERP: Event-related potential
BCI: Brain-computer interface
P300: Positive spike at 300 milliseconds
N200: Negative spike at 200 milliseconds
